# Tubular adenoma of the breast: radiological and ultrasound findings

**DOI:** 10.1590/0100-3984.2017.0012

**Published:** 2018

**Authors:** Rodrigo Amaral Rodrigues, Carmen Lúcia Arantes Pereira Azevedo, Maria Célia Resende Djahjah, Talita Siemann Santos Pereira

**Affiliations:** 1 Hospital Universitário Clementino Fraga Filho da Universidade Federal do Rio de Janeiro (HUCFF-UFRJ), Rio de Janeiro, RJ, Brazil.

*Dear Editor,*

**Figure 1:**
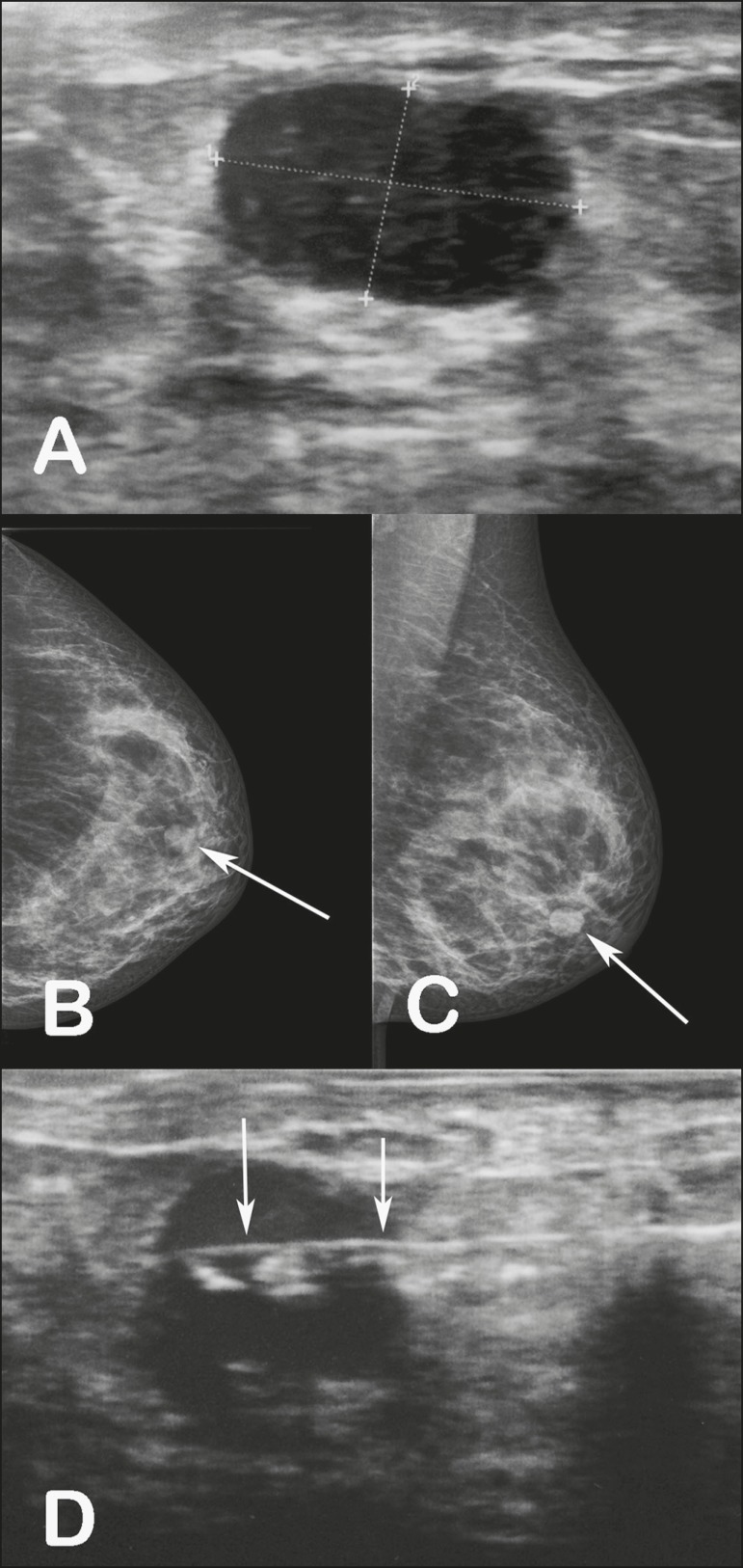
A: Ultrasound showing a well-circumscribed, hypoechoic nodule, measuring 12 × 8 mm, in the lower outer quadrant of the left breast. B,C: Mammography, in craniocaudal and mediolateral oblique views, respectively, showing a well-circumscribed, isodense nodule, measuring 12 mm, in the lower outer quadrant of the left breast. D: Ultrasound-guided percutaneous core biopsy of the nodule. The arrows indicate the needle within the nodule.

A 34-year-old female patient presented to the breast diagnostic clinic with a palpable nodule in the lower outer quadrant of the left breast. Ultrasound showed a solid, hypoechoic, well-circumscribed nodule, measuring 12 × 8 mm, in the lower outer quadrant of the left breast (Figure 1A). The nodule had not been visible on an ultrasound examination performed a year earlier. Mammography revealed a well-circumscribed, isodense nodule, measuring 12 mm, in the lower outer quadrant of the left breast (Figures 1B and 1C), corresponding to the lesion observed on ultrasound. A percutaneous core biopsy was performed (Figure 1D), the histopathological analysis of which showed tubular adenoma of the breast, consistent with the radiological and ultrasound findings. Therefore, it was recommended that the patient undergo another ultrasound examination in six months and be followed in the breast disease department.

Tubular adenoma of the breast is a rare benign epithelial tumor of the breast that has not been widely studied; the World Health Organization defines it as a “benign, usually round, nodules formed by a compact proliferation of tubular structures composed of typical epithelial and myoepithelial cell layers”^(1,2)^.

Although four cases of malignant transformation of tubular adenoma have been reported in the literature, studies indicate that there is no high risk for carcinoma^(1)^. Tubular adenoma accounts for 0.13-1.7% of all benign neoplasms of the breast^(3)^. Although the size of the tumor ranges from 1 cm to 7.5 cm, it rarely exceeds 5 cm^(4)^. The vast majority of cases are in young women, and the disease is much more rare in postmenopausal women, 90% of the patients being under 40 years of age (mean, 31 years). Nevertheless, there have been reports of its occurrence in males. It has not been found to be associated with oral contraceptive use or pregnancy^(4,5)^. It is considered a variant of fibroadenoma, appearing in the same clinical context and with overlapping imaging characteristics^(6)^. It can be difficult to make the histological differentiation between tubular adenoma and fibroadenoma if the tubular adenoma has a relatively abundant stromal component or the fibroadenoma shows significant proliferation of small ducts^(1)^.

Clinically, tubular adenomas of the breast can be asymptomatic, occasionally being detected on mammography or physical examination as a palpable nodule that gradually increases in size^(4)^. On mammography and ultrasound, these tumors have the appearance of noncalcified fibroadenomas^(7)^. On mammography, the lesions typically appear as well-circumscribed nodules, with no evidence of calcifications. However, in older patients, punctate or irregular calcifications can be observed, findings that justify a biopsy to exclude malignant neoplasm of the breast. Occasionally, mammography shows lesions with ill-defined margins. On ultrasound, the tumors are generally described as hypoechoic, well-circumscribed nodules. Noncalcified tubular adenomas generally have a relatively homogeneous internal texture and may have posterior acoustic reinforcement^(4)^. Other differential diagnoses that should be included are ductal adenomas, lactating adenoma, gestational hyperplasia, and ductal carcinoma^(8)^. Sengupta et al.^(3)^, analyzing 32 confirmed cases of tubular adenoma, concluded that, although radiological and cytological studies can distinguish between benign and malignant lesions, the final diagnosis depends on the histopathology^(3)^.
